# Identification of EMS-induced sesame (*Sesamum indicum L*.) mutants with improved low-temperature tolerance during germination and early seedling growth

**DOI:** 10.3389/fpls.2026.1849760

**Published:** 2026-06-17

**Authors:** Nouhayla Tahri, Boutaina Louafi, Mohamed Kouighat, Karima Mikou, Abdelghani Nabloussi

**Affiliations:** 1 Department of Life Sciences, Laboratory of Functional Ecology and Environmental Engineering, Faculty of Sciences and Techniques, Sidi Mohamed Ben Abdellah University, Fez, Morocco; 2 Research Unit of Plant Breeding and Plant Genetic Resources Conservation, National Institute of Agricultural Research, Regional Agricultural Research Center of Meknes, Meknes, Morocco

**Keywords:** climate adaptation, cold tolerance, early seedling growth, germination, Morocco, seedling vigor

## Abstract

Sesame (*Sesamum indicum* L.) is a valuable oilseed crop in Morocco, but its cultivation is strictly constrained by sensitivity to low temperatures during early-season sowing. Traditionally sown in June, advancing the sowing date to April would allow farmers to capitalize on residual spring rainfall, significantly reduce irrigation dependency, and improve the crop productivity. However, this strategy exposes germinating seeds to sub-optimal soil temperatures (10-15 °C night minima). Mutation breeding via ethyl methanesulfonate (EMS) offers a powerful approach to generate novel genetic variability for abiotic stress tolerance. This study aimed to identify EMS-induced sesame mutants exhibiting superior germination and early seedling growth performance under progressive low-temperature stress conditions. Germination and seedling growth traits were assessed under controlled *in vitro* conditions. Low temperature stress induced highly significant genotypic variation (p<0.001) for all traits except for root and plumule length. At the coldest regime (24 °C/12 °C), mutants ML2–37 and ML2–72 maintained significantly higher germination percentages compared to the cold-sensitive mutant US1-2. The tolerant mutants exhibited faster germination rates, shorter mean germination times, superior root-shoot ratios, and higher seedling vigor indices. The mutants ML2–37 and ML2-72, demonstrating promising low-temperature tolerance during the critical germination phase, represent valuable genetic resources for developing early-sowing, climate-resilient sesame cultivars. However, since the present study was conducted exclusively under controlled laboratory conditions, the findings should be supported by an extensive field validation across multiple sites and seasons in order to confirm their agronomic performance and yield stability under real early-spring sowing conditions.

## 1 Introduction

Sesame (*Sesamum indicum* L.) is one of the oldest oilseed crops known to humanity, cultivated for over 5,000 years. Often referred to as the “queen of oilseeds”, it is prized for its high oil content (48-54%), high oil and protein quality, and richness in natural antioxidants such as sesamin and sesamolin, which confer remarkable stability and health benefits (Bedigian and Harlan, 1986; Rizki et al., 2015). In Morocco, sesame plays a significant role in the agricultural economy of the Tadla (Beni Mellal) region, where it serves as a crucial cash crop for smallholder farmers. However, despite its long history and nutritional value, national productivity remains constrained by traditional management practices and environmental limitations (Kouighat et al., 2022).

While sesame exhibits moderate tolerance to drought and heat stress adaptations well suited to semi-arid environments, the crop is notoriously sensitive to low temperatures, particularly during the critical stages of germination and early seedling establishment (El Harfi et al., 2016). Cold stress disrupts metabolic processes, slows enzymatic activity, impairs membrane integrity through phase transition of lipids, and severely reduces water uptake (Abbas et al., 2025). Currently, sesame in Morocco is sown in early June, following the harvest of winter cereals. This calendar places the crop in the middle of the hot, dry summer, necessitating intensive irrigation to meet evapotranspiration demands. Consequently, sesame must compete for scarce water resources with high-priority crops such as citrus and vegetables, often relegating it to secondary status.

Advancing the sowing date from June to April represents a strategic agronomic shift to alleviate these constraints. Early sowing would allow the crop to capitalize on residual spring rainfall and soil moisture, significantly reducing dependency on irrigation water, avoiding peak summer heat during sensitive reproductive stages, and thus improving its productivity. However, this strategy presents a major physiological hurdle: soil temperatures in Morocco’s main production zones (Tadla, Saïs) during April often drop to 12-15 °C at night, well below the optimal range for sesame germination (25-30 °C). Under these sub-optimal thermal conditions, standard cultivars suffer from delayed emergence, poor stand uniformity, and increased susceptibility to soil-borne pathogens. Therefore, the development of cold-tolerant germplasm capable of vigorous germination under cool spring conditions is a prerequisite for expanding the sesame sowing window.

Chemical mutagenesis using ethyl methanesulfonate (EMS) has emerged as a powerful and efficient tool to generate novel genetic variation in crops with narrow genetic bases, such as sesame. Unlike transgenic approaches, mutation breeding is widely accepted and not subject to strict regulatory limitations. EMS induces point mutations (G/C to A/T transitions) distributed randomly throughout the genome, potentially creating alleles that confer tolerance to abiotic stresses (Greene et al., 2003). Previous successful applications of EMS mutagenesis in sesame have resulted in the identification of mutants with enhanced drought tolerance (Kouighat et al., 2021), altered plant architecture, and improved yield components (Kouighat et al., 2020).

Research on sesame responses to low temperatures during germination is still limited. Early studies mainly described the general sensitivity of the species to cold stress (Smilde, 1960), while more recent investigations focused primarily on severe cold conditions (10-16 °C) and associated physiological responses (Abbas et al., 2025). To date, little attention has been paid to moderate low-temperature regimes representative of actual early spring sowing conditions in Mediterranean environments. Moreover, no previous study has specifically evaluated EMS-induced sesame mutants under progressive temperature regimes designed to realistically simulate Moroccan spring conditions. This represents a significant research gap and highlights the originality of the present work.

Therefore, the novelty of this study lies in the systematic evaluation of EMS-induced sesame mutants for germination and early seedling performance under realistic spring temperature scenarios relevant to Morocco’s sesame-growing regions. Unlike previous studies conducted under extreme or generalized stress conditions, the present work specifically addresses moderate nocturnal cold stress that farmers are likely to encounter under early sowing systems. The study also integrates agronomic and physiological screening approaches to identify elite mutant lines with potential for climate-resilient sesame production.

The present study aimed to screen and identify EMS-induced sesame mutants exhibiting improved performance under low-temperature stress during germination and early seedling growth. The specific objectives were to: (1) evaluate germination dynamics (percentage, rate, time) of promising mutant lines and their parental line under progressive temperature regimes simulating Moroccan spring conditions; (2) assess early seedling vigor and developmental plasticity (root/shoot growth) under cold stress; (3) identify elite, cold-tolerant mutants suitable for field validation in early-sowing systems; and (4) discuss the potential physiological mechanisms underlying differential cold tolerance. The findings of this study are expected to contribute to breeding strategies aimed at improving climate resilience and promoting sustainable sesame production in semi-arid environments.

## 2 Materials and methods

### 2.1 Plant material

The plant material used in this study consisted of six sesame genotypes: the local Moroccan cultivar ‘ML13’ (wild-type parent) and five stable mutant lines (M5) derived from EMS mutagenesis. The mutants were selected from the M3 and M4 generations based on stable phenotypic expression and preliminary screening for abiotic stress tolerance (Kouighat et al., 2020, 2022). The mutant line US1–2 was originally derived from parental line US06 used for mutagenesis. However, the parental line US06 was not included in the present study, as the experimental design specifically aimed to compare the responses of the selected mutant lines with those of the Moroccan cultivar ML13 under low-temperature stress conditions. All seed samples were stored in a cold chamber under controlled conditions, ensuring uniformity of storage conditions prior to experimentation. The characteristics of the six genotypes are summarized in **Table 1**.

**Table 1:** Main phenotypic, morphological, and imbibition characteristics of the studied sesame genotypes.

Genotype	Origin/generation	Key characteristics & selection criteria	Seed length (mm) mean ± SD (min–max)	Seed weight (mm) mean ± SD (min–max)	L/W ratio mean ± SD	Seed shape	Mean seed weight (mg) mean ± SD	Imbibition percentage (%)
ML13	Morocco, Parent (M0)	Wild-type, beige seeds, single capsule/axil, locally adapted	3.30 ± 0.60 (2.5–4.0)	1.78 ± 0.23 (1.5–2.0)	1.89 ± 0.52	Isodiametric	3.145 ± 0.68	36.0
ML2-5	ML13 mutant, M5	Brown seeds, tall stature, large capsules	3.62 ± 0.40 (3.0–4.0)	1.30 ± 0.44 (1.0–2.0)	2.66 ± 0.64	Oblong	3.596 ± 0.41	79.5
ML2-10	ML13 mutant, M5	Brown seeds, high branching, profuse small capsules	3.14 ± 0.54 (2.5–4.0)	2.00 ± 0.00 (2.0–2.0)	1.57 ± 0.27	Elliptic	2.469 ± 0.57	58.0
ML2-37	ML13 mutant, M5	Beige seeds, thick leathery leaves, drought tolerance	2.90 ± 0.22 (2.5–3.0)	2.00 ± 0.00 (2.0–2.0)	1.45 ± 0.11	Ovoid	2.803 ± 0.36	44.0
ML2-72	ML13 mutant, M5	Light brown seeds, late flowering, high 1000-seed weight	2.80 ± 0.44 (2.0–3.0)	1.40 ± 0.54 (1.0–2.0)	2.30 ± 0.97	Oblong	2.784 ± 0.56	49.0
US1-2	US06 mutant, M5	White seeds, high capsule number, high yield potential	3.00 ± 0.00 (3.0–3.0)	1.60 ± 0.54 (1.0–2.0)	2.10 ± 0.82	Oblong	2.804 ± 0.63	38.5

L/W, Length-to-weight ratio; SD, standard deviation.

### 2.2 Seed treatment under low temperature stress

The experiment was conducted at the Laboratory of Functional Ecology and Environmental Engineering (FST-Fes) in collaboration with INRA-Meknes. To evaluate cold tolerance, seeds were subjected to four distinct temperature regimes with a 14h light/10h dark photoperiod. Petri dishes were randomly repositioned daily within the incubator to minimize the effect of temperature gradients. Optimal conditions for sesame germination (30 °C/18 °C, Control), mild stress (28 °C/16 °C), moderate stress (26 °C/14 °C), and severe stress or coldest regime (24 °C/12 °C). These regimes were specifically designed to simulate the progressive cold stress characteristic of early-to-mid April period in Morocco’s major sesame production zones. Meteorological data (2010–2020 averages) for the Tadla and Saïs regions indicate mean April temperatures of 18-25 °C during the day and 12-15 °C at night. The coldest treatment (24 °C/12 °C) represents the early-spring temperature range, while milder treatments capture the transitional conditions between sub-optimal and optimal environments.

Seeds were surface-sterilized with 5% sodium hypochlorite for 5 min and then thoroughly rinsed three times with distilled water to ensure complete removal of sodium hypochlorite and prevent fungal contamination. For each treatment, 20 seeds per genotype were placed in 9 cm diameter Petri dishes containing double-layered Whatman filter paper moistened with 2 mL of distilled water. Three Petri dishes were used as biological replicates for each genotype × temperature treatment combination. Germination tests were performed in a temperature and humidity controlled Memmert incubator. The Petri dishes were incubated in a controlled growth chamber under the specified temperature regimes with a 14 h light/10 h dark photoperiod. Relative humidity was maintained at 70% throughout the experiment. Petri dishes were randomly arranged within the incubator and periodically repositioned during the experiment to minimize positional effects. Moisture levels were maintained throughout the experiment by adding distilled water when necessary. The sample size of 20 seeds per replicate (60 seeds total per genotype-treatment combination) was determined based on preliminary trials and resource constraints. Statistical power analysis indicated this size provides a power >0.80 to detect differences ≥15% in germination percentage at α=0.05. While this is below ISTA standards (4 replicates × 50 seeds), it balances precision with the feasibility of screening multiple genotypes under controlled incubator conditions.

The germination test duration was set to 10 days. This duration allows for the full expression of differential germination responses under temperature stress, capturing both the rapid germination of tolerant genotypes and the delayed emergence of sensitive ones (Bakhshandeh et al., 2017; Kouighat et al., 2021).

### 2.3 Experimental design

The experiment followed a Completely Randomized Design (CRD) with two factors: Genotype (6 levels) and Temperature Regime (4 levels). Each combination was replicated three times, resulting in a total of 72 experimental units (Petri dishes). To maintain uniform moisture, 2 mL of distilled water was added to the dishes every two days. Seeds were considered germinated when the radicle protruded at least 2 mm.

### 2.4 Parameters measured

Germination counts were recorded every 48 hours. The following parameters were calculated:

Germination Percentage (GP): Calculated on day 10 as.


$$GP=\frac{N_{10}}{20}\times \;100$$

where N_10_ is the number of germinated seeds.

Germination Rate (GR): Calculated as


$$GR=\frac{\sum \left(G_{i}-G_{i-1}\right)}{i}$$

Where Gi is the cumulative number of germinated seeds on day i. Higher values indicate faster germination.

Mean Germination Time (MGT): Calculated as:


$$MGT=\frac{(\sum \left(ni\times di\right)}{\left(\sum ni\right)}$$

Where ni is the number of seeds germinated on day di (Ullah et al., 2022).

Seedling Growth:

On the 10th day, 10 randomly selected seedlings from each Petri dish were individually measured for Root Length (RL), Shoot Length (SL), and Plumule Length (PL) using a digital caliper. Three Petri dishes were used as biological replicates per treatment. The mean value of the 10 seedlings from each Petri dish was calculated and used for statistical analysis, with each Petri dish considered as one experimental unit.

Root-Shoot Ratio (RSR): Calculated as


$$RSR=\frac{RL}{SL}$$

Seedling Vigor Index (SVI): Calculated as:


$$SVI=\frac{Seedling\;Length\;\times \;GP}{100}$$

(Sadeghi et al., 2011).

### 2.5 Statistical analysis

Data were subjected to a two-way Analysis of Variance (ANOVA) using SPSS software (version 25.0) and GraphPad Prism 9, with Genotype and Stress considered as fixed effects. Percentage data were analyzed directly without transformation, as the assumptions of ANOVA were satisfied. When the F-test was significant, means were compared using Duncan’s Multiple Range Test (DMRT) at p< 0.05. Significant differences among means are indicated by different letters in the figures. Error bars in the graphs represent standard deviation (SD). Exact p-values are provided where appropriate.

The Coefficient of Variation (CV%) was calculated as (Standard Deviation/Mean) × 100 to assess experimental precision. Detailed genotype discrimination and multiple comparison analyses were mainly emphasized under the coldest regime (24/12 °C), considered the most relevant condition for identifying potentially cold-tolerant genotypes adapted to early spring sowing conditions in Morocco.

## 3 Results

### 3.1 Low temperature effects on germination parameters

The analysis of variance (ANOVA) revealed that low-temperature stress exerted a highly significant influence (p< 0.001) on all measured germination and seedling traits (**Table 2**). The genotype effect was also highly significant for germination rate (GR) and percentage (GP), mean germination time (MGT), shoot length (SL), root-shoot ratio (RSR), and seedling vigor index (SVI), indicating substantial genetic variability among the studied genotypes. Nevertheless, these genotypes are statistically comparable for root length (RL) and plumule length (PL). On the other hand, the interaction between genotype and low-temperature stress was significant for all traits, demonstrating that the genotypes responded differentially and specifically to the temperature regimes.

**Table 2:** Analysis of variance (mean squares) and coefficient of variation for seed germination traits of six sesame genotypes under low temperature stress.

Source	DF	GR	MGT	GP	RL	SL	SVI	RSR	PL
Genotype (G)	5	10.13***	0.01***	369.5***	0.78ns	0.62**	26849**	0.48***	0.60ns
Low-temperature Stress (S)	3	46.73***	0.04***	152.2***	29.77***	8.34***	281976***	2.94***	41.22***
G × S	15	2.92***	0.01***	99.14***	2.00***	0.79***	57803***	0.13*	4.27***
Error	48	0.16	0.00	1.74	0.36	0.17	5698	0.06	0.48
**CV%**	–	**12.50%**	**8.30%**	**4.20%**	**15.80%**	**13.40%**	**18.70%**	**11.90%**	**14.20%**

DF, degrees of freedom; GP, percentage of germination; GR, germination rate; MGT, mean germination time; SVI, seedling vigor index; RL, root length; SL, shoot length; PL, plumule length; RSR, root length/shoot ratio. ns, non-significant; *p ≤ 0.05; **p ≤ 0.01; and ***p ≤ 0.001.

Bold values indicate the coefficients of variation (CV%) for each trait.

### 3.1.1 Germination percentage

**Figure 1:**
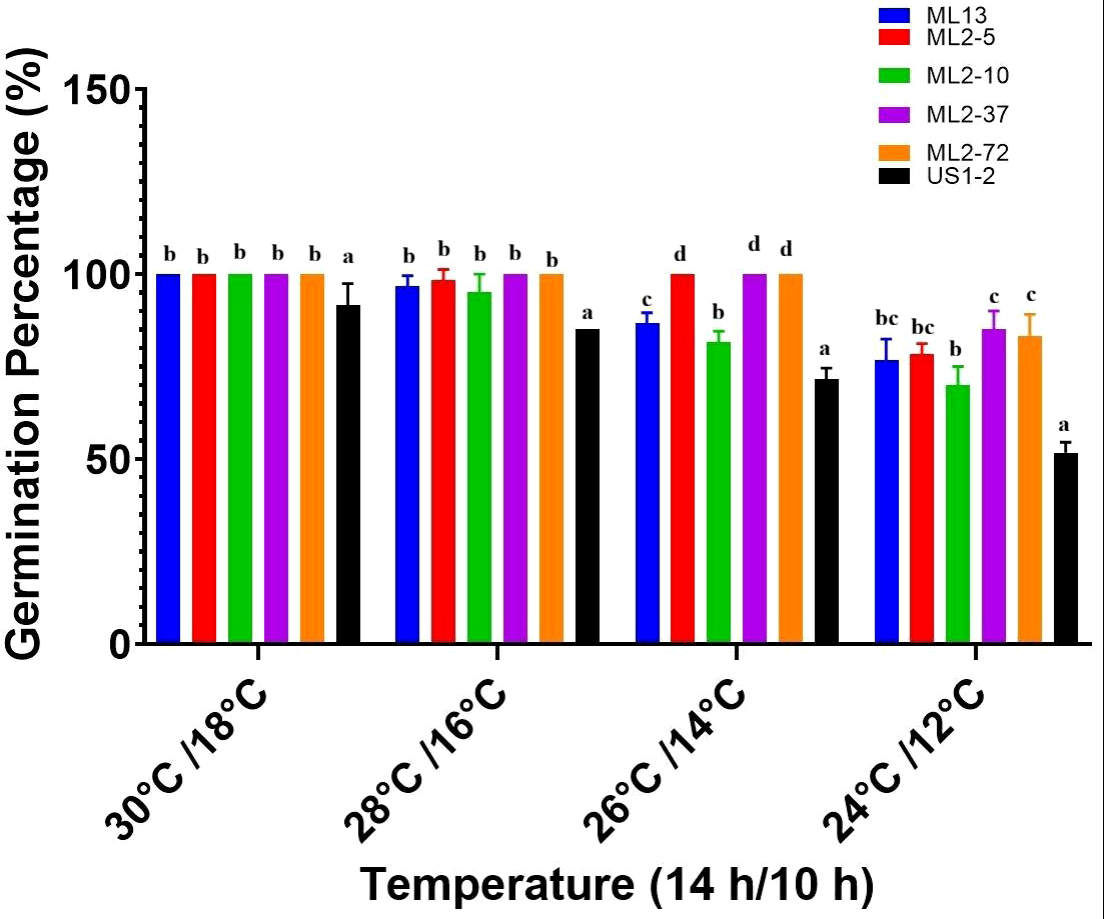
Effect of progressive low-temperature stress on germination percentage (GP) in six sesame genotypes evaluated under four temperature regimes: 30 °C/18 °C (control), 28 °C/16 °C, 26 °C/14 °C, and 24 °C/12 °C (day/night;14h light/10h dark photoperiod). Data are presented as mean ± standard error of the mean (SEM). Values with different alphabetical superscripts are significantly different (p ≤ 0.05) according to DMRT.

As shown in **Figure 1**, the germination percentage (GP) was important in all the genotypes under control temperature conditions (30 °C/18 °C), with values reaching 100%, except the mutant US1–2 displaying slightly lower GP, reaching a value around 90%. These results indicate that, in the absence of cold stress, all mutants, along with the parental cultivar ML13, maintain excellent germination capacity comparable to optimal conditions. Under mild low temperature stress (28 °C/16 °C), the values of germination percentage remained high and close to the control. However, for moderate stress (26 °C/14 °C), differences among the genotypes became more visible, with ML13, ML2–10 and US1–2 exhibiting a considerable decrease in the GP values (85%, 80%, and 75% respectively), while ML2-5, ML2-72, and ML2–37 continued showing high stability, maintaining GP at 100%. Under coldest regime (24 °C/12 °C), a clear separation in tolerance among genotypes was observed. The mutant ML2–37 showed the strongest resilience, followed by ML2-72 (GP close to 85%), while ML2–10 displayed more substantial decline, reaching approximately 75%. The mutant US1–2 showed its highest sensitivity to the coldest regime, with the lowest GP value (close to 55%), compared to the other genotypes.

#### 3.1.2 Germination rate and mean germination time

**Figure 2:**
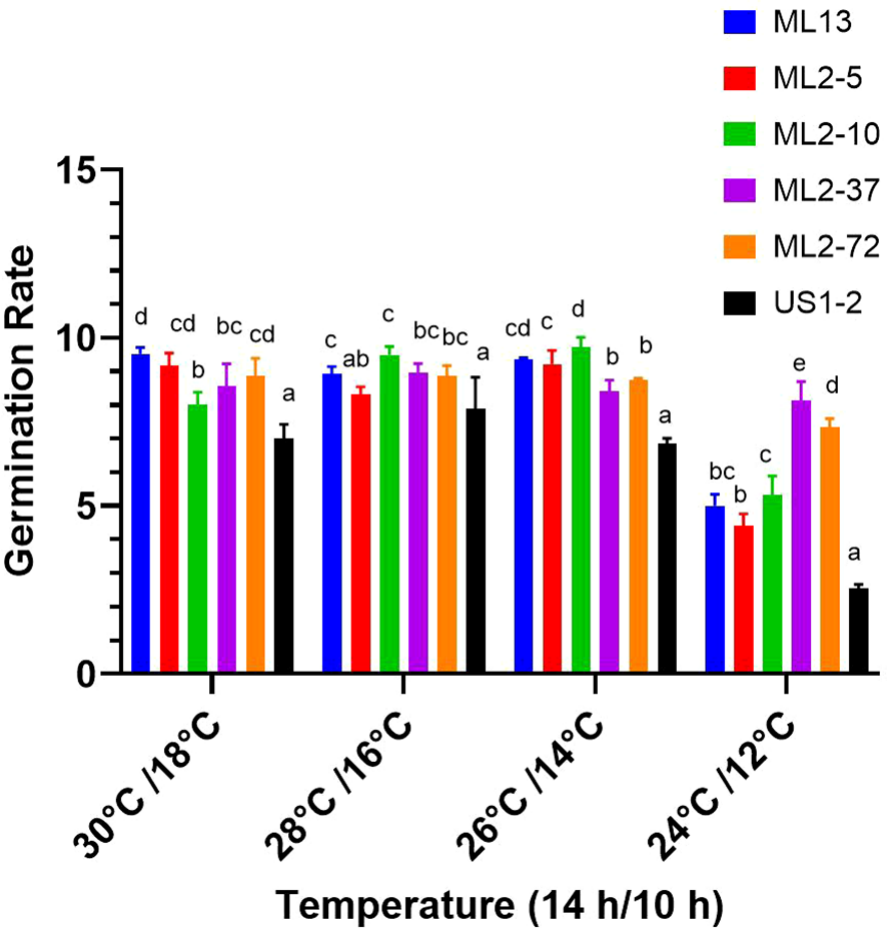
Effect of low temperature stress on germination rate (GR) in six sesame genotypes evaluated under four temperature regimes: 30 °C/18 °C (control), 28 °C/16 °C, 26 °C/14 °C, and 24 °C/12 °C (day/night, 14h light/10h dark photoperiod). Data are presented as mean ± standard error of the mean (SEM). Values with different alphabetical superscripts are significantly different (p ≤ 0.05) according to DMRT.

**Figure 2** shows the effect of low temperature stress on germination rate (GR). Under control conditions (30 °C/18 °C), germination rate was relatively high for all the genotypes, ranging from 7 to 9.50. As the temperature decreased (28 °C/16 °C), ML13 and ML2–5 reflected a gradual reduction, confirming that mild cold stress negatively affects their germination dynamics. On the other hand, the other genotypes showed a slight increase compared to the control, with ML2–10 having relatively higher GR value, indicating better adaptation to such conditions. At moderate stress (26 °C/14C), most genotypes still preserved acceptable germination rates, with ML2-10, ML2-5, and ML2–13 showing the highest performance (~9-10), while US1–2 having the lowest GR (~7), reflecting its sensitivity. At the severe low temperature level (24 °C/12 °C), germination rate decreased in all the studied genotypes, with US1–2 having, once again, the lowest value (< 3). However, the genotypes ML2–37 and ML2–72 are the most likely tolerant to this cold stress level, maintaining the highest value (7 and 6.50 respectively).

**Figure 3:**
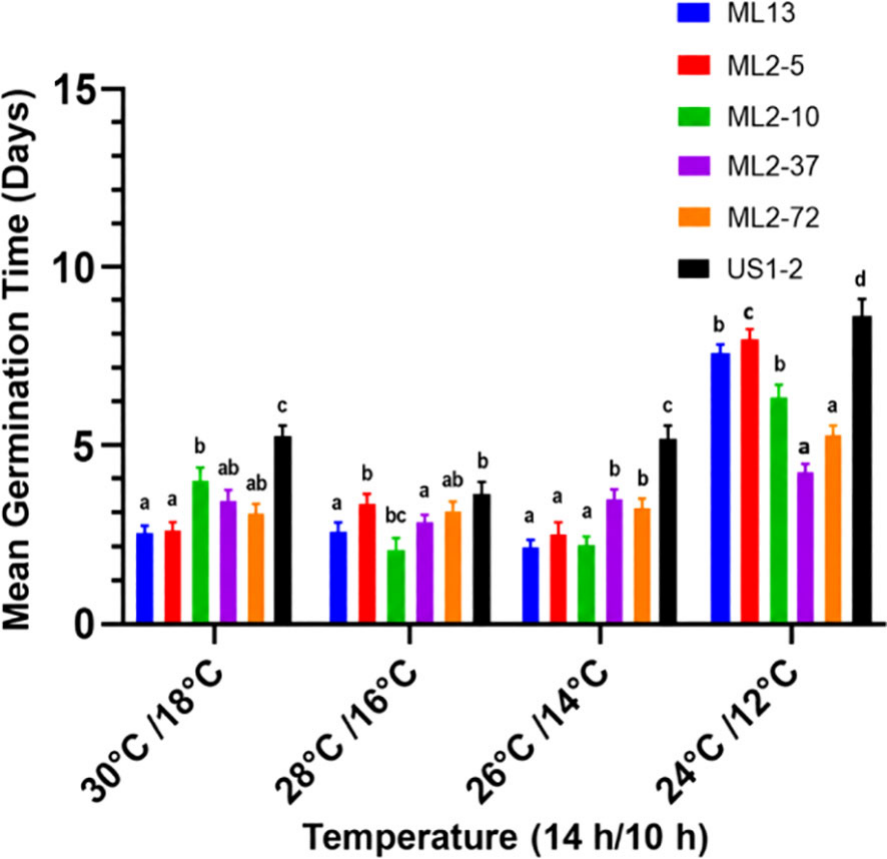
Effect of progressive low-temperature stress on mean germination time (MGT) in six sesame genotypes evaluated under four temperature regimes: 30 °C/18 °C (control), 28 °C/16 °C, 26 °C/14 °C, and 24 °C/12 °C (day/night, 14h light/10h dark photoperiod). Data are presented as mean ± standard error of the mean (SEM). Values with different alphabetical superscripts are significantly different (p ≤ 0.05) according to DMRT.

Regarding mean germination time, presented in **Figure 3**, low and relatively stable values across all the genotypes were observed during (30 °C/18 °C), indicating rapid and efficient germination. Under intermediate temperatures (28 °C/16 °C and 26 °C/14 °C), MGT values were close to the control treatment in all genotypes, reflecting their adaptation to such thermal conditions. In contrast, the coldest regime (24 °C/12 °C) caused a sharp rise in MGT for all genotypes, with US1–2 having the highest drastic increase, demonstrating extreme sensitivity and slowest germination. In contrast, ML2–37 and ML2–72 remained the most resilient, highlighting their capacity to maintain a rapid germination despite low-temperature stress.

#### 3.1.3 Seedling growth and vigor

**Figure 4:**
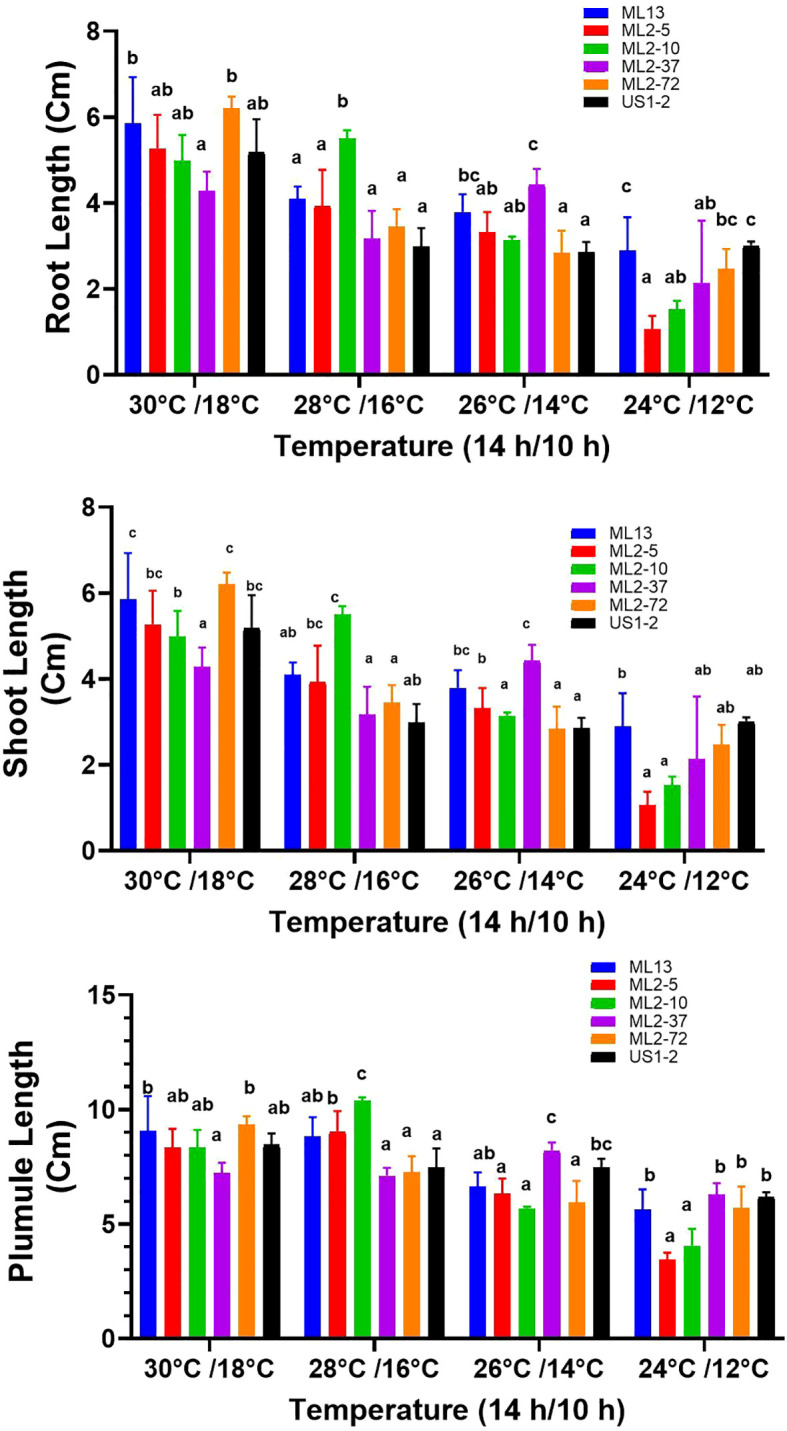
Effect of progressive low-temperature stress on seedling growth parameters: root length (RL), shoot length (SL), and plumule length (PL) in six sesame genotypes evaluated under four temperature regimes: 30 °C/18 °C (control), 28 °C/16 °C, 26 °C/14 °C, and 24 °C/12 °C (day/night, 14h light/10h dark photoperiod). Data are presented as mean ± standard error of the mean (SEM). Values with different alphabetical superscripts are significantly different (p ≤ 0.05) according to DMRT.

In general, the lower the temperature, the more pronounced the decline across all measured parameters, root length (RL), shoot length (SL), and plumule length (PL). At the control temperature (30 °C/18 °C), the investigated genotypes showed a vigorous growth, with ML2–72 and ML13 having the longest RL, SL, and PL values (**Figure 4**). Under moderate conditions (28 °C/16 °C and 26 °C/14 °C), all these parameters were progressively reduced, indicating an inhibitory cold-effect on cell elongation and early seedling development, across all the genotypes, except for ML2-10. Under severe stress (24 °C/12 °C), all genotypes were affected, exhibiting a marked decrease in their RL, SL, and PL. The mutant ML2–5 showed the lowest RL, SL, and PL, while ML13 and US1–2 maintained higher RL, SL, compared to the other genotypes under this stress level. Nevertheless, the genotypes ML2–37 and ML2–72 were found to be statistically comparable with ML13 and US1–2 for plumule and shoot length.

**Figure 5:**
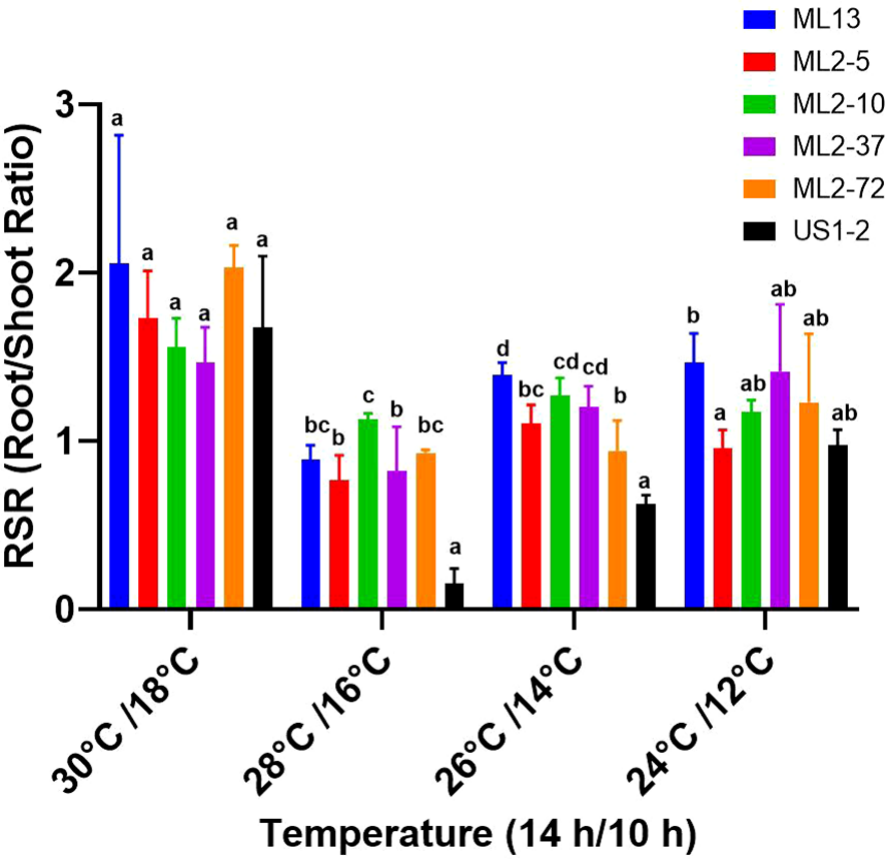
Effect of progressive low-temperature stress on root-shoot ratio (RSR) in six sesame genotypes evaluated under four temperature regimes: 30 °C/18 °C (control), 28 °C/16 °C, 26 °C/14 °C, and 24 °C/12 °C (day/night, 14h light/10h dark photoperiod). Data are presented as mean ± standard error of the mean (SEM). Values with different alphabetical superscripts are significantly different (p ≤ 0.05) according to DMRT.

The root-to-shoot ratio (RSR) varied among the six sesame genotypes under decreasing temperature conditions, indicating differential responses to low-temperature stress (**Figure 5**). Under control conditions (30 °C/18 °C), ML13 and ML2–72 exhibited the highest RSR values, whereas ML2–37 showed the lowest value. At 28 °C/16 °C, a reduction in RSR was observed in all genotypes, particularly in US1-2, while ML2–10 maintained relatively higher RSR values. Under moderate low-temperature stress (26 °C/14 °C), ML13, ML2-10, and ML2–37 recorded comparatively higher RSR values, whereas US1–2 showed a marked decline compared with the control treatment.

Under the coldest regime (24 °C/12 °C), ML13 and ML2–37 maintained relatively higher RSR values compared with the other genotypes. Similarly, ML2–5 showed moderate stability despite lower numerical values. Overall, ML13, ML2-10, and ML2–37 tended to maintain relatively stable RSR values across temperature regimes, suggesting better adaptation to low-temperature conditions. In contrast, US1–2 consistently exhibited the lowest RSR values across all stress treatments, confirming its sensitivity to low-temperature stress.

**Figure 6:**
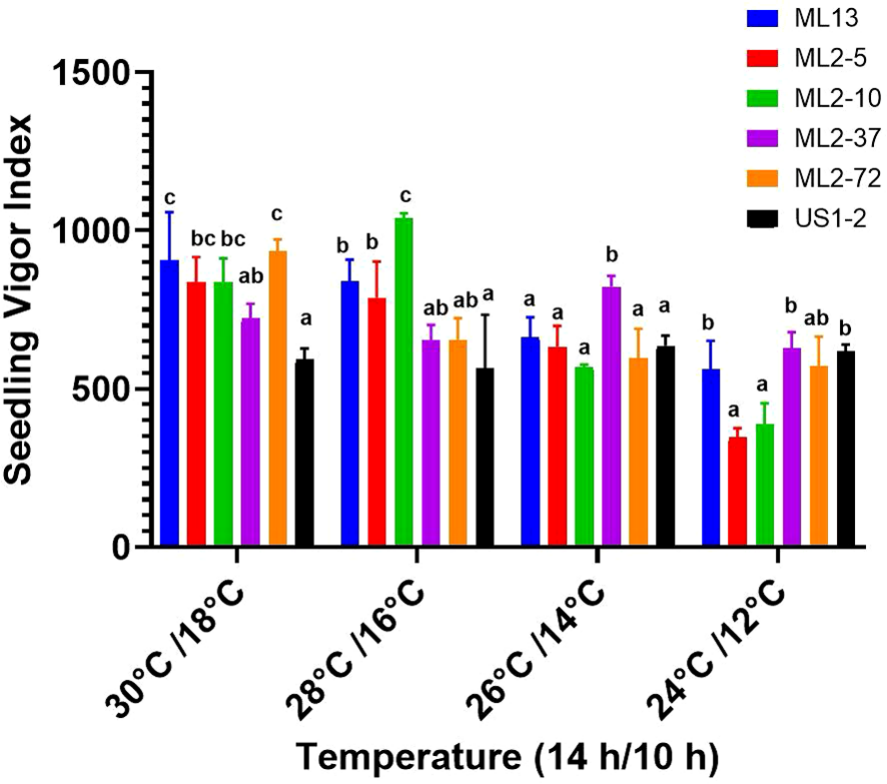
Effect of progressive low-temperature stress on seedling vigor index (SVI) in six sesame genotypes evaluated under four temperature regimes: 30 °C/18 °C (control), 28 °C/16 °C, 26 °C/14 °C, and 24 °C/12 °C (day/night, 14h light/10h dark photoperiod). Data are presented as mean ± standard error of the mean (SEM). Values with different alphabetical superscripts are significantly different (p ≤ 0.05) according to DMRT.

Overall, seedling vigor index (SVI) declined progressively as temperature decreased, with some exceptions in marked genotype variations and differences in cold tolerance (**Figure 6**). Under control conditions (30 °C/18 °C), the genotypes ML2-72, and ML13 exhibited the strongest performance (highest SVI), while the genotype US1–2 showed the lowest vigor. At mild temperature stress (28 °C/16 °C), SVI decreased for all the genotypes, except for ML2–10 with the highest SVI, indicating better performance. Under moderate temperature stress conditions (26 °C/14 °C), one could observe a notable decline across genotypes. Nevertheless, the mutant line ML2–37 exhibited the highest SVI, suggesting better resilience at intermediate cold level. Under the lowest temperature (24 °C/12 °C), SVI reached minimum values in all genotypes, confirming the strong impact of such a stress on seedling growth. However, ML13, ML2–37 and ML2–72 maintained higher SVI than the rest of genotypes, suggesting their better performance under cold stress conditions. In contrast, the genotypes ML2–5 and ML2–10 experienced the most drastic decline in SVI, indicating their sensitivity to low temperatures, compared to the other genotypes.

### 3.2 Results of multi-traits analysis under cold stress

#### 3.2.1 Correlation among the studied traits

**Figure 7:**
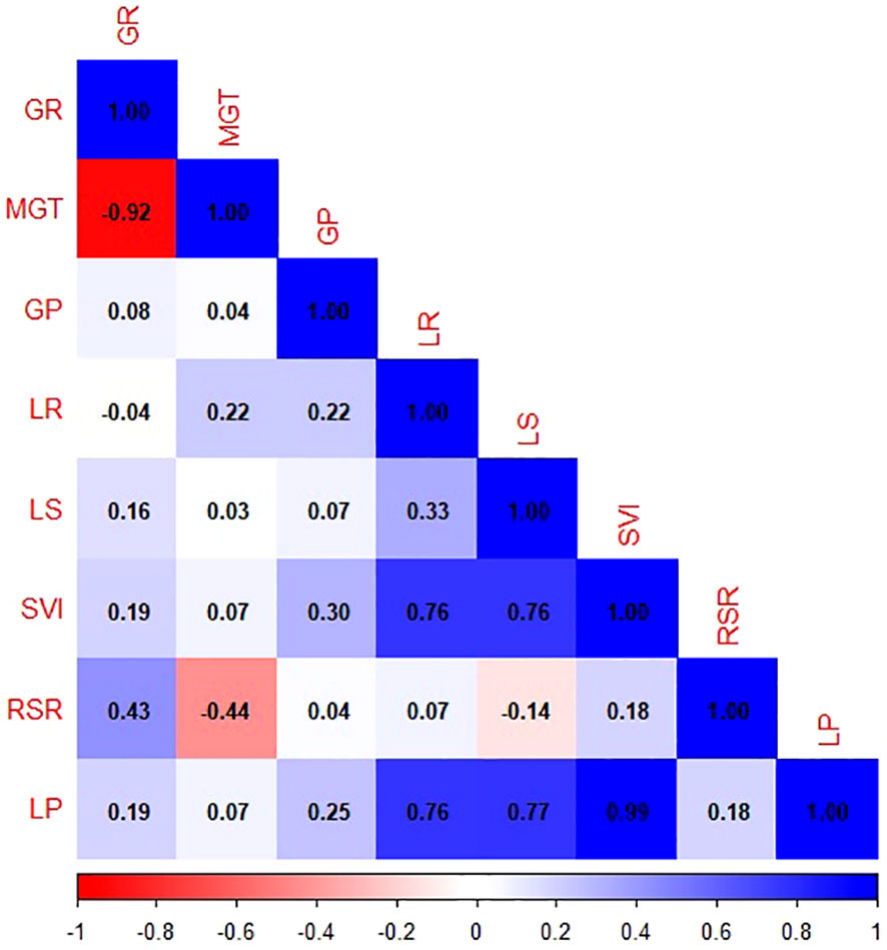
Correlation analysis of measured traits in six sesame genotypes during germination and early seedling growth at severe low temperature stress (24 °C/12 °C) during the germination and seedling growth stage. MGT, Mean Germination Time; GR, Germination Rate GP, Germination Percentage; SL, Shoot Length; RL, Root Length; PL, Plumule Length; RSR, Root-to-Shoot Ratio; SVI, Seedling Vigor Index.

**Figure 7** reveals several strong associations between the studied variables. Strong negative correlation showed between germination rate (GR) and mean germination time (MGT) (r = -0.92), indicating that genotypes with faster germination are low in mean germination time. In addition, very strong positive correlations are observed between seed vigor index (SVI) and root length (RL) (r = 0.76), as well as between SVI and plumule length (PL) (r = 0.99).

Similarly, plumule length (PL) is strongly correlated with root length (RL) (r = 0.76) and SVI (r = 0.99), reflecting a close association between seedling growth and seed vigor. These relationships indicate that genotypes with higher seed vigor also develop longer roots and plumules.

Conversely, the strong negative correlation between GR and MGT confirms that rapid germination is a key factor associated with improved germination performance.

Overall, these correlations highlight the importance of GP, RL, SVI, SL, and PL as key traits associated with improved germination performance and early seedling development under low temperature stress.

#### 3.2.2 Genotypes grouping

**Figure 8:**
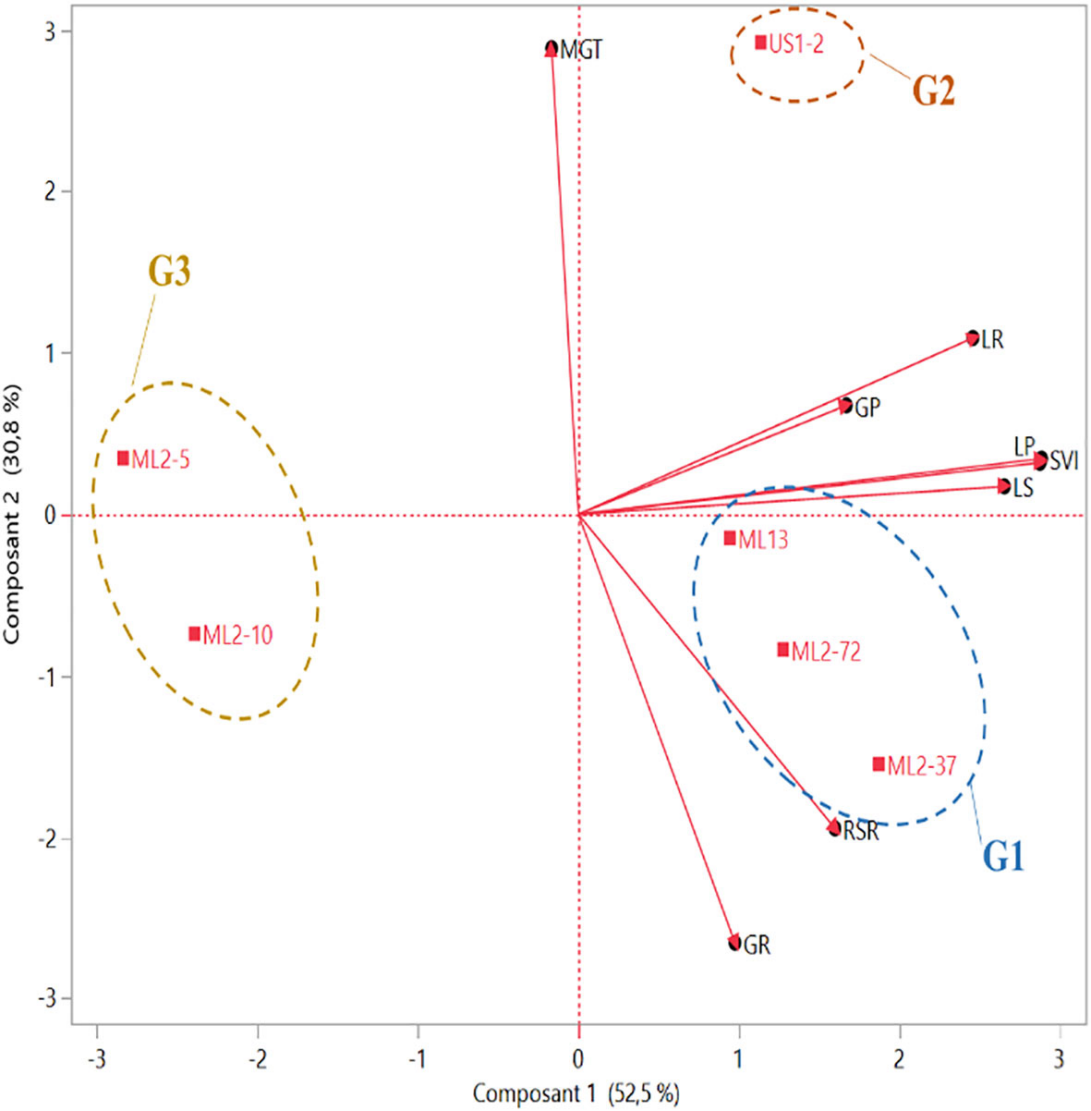
Biplot representing the diversity of six sesame genotypes at severe low temperature stress (24 °C/12 °C) during the germination and seedling growth stage. The greater values of PC1 and PC2 indicate which genotypes were more tolerant to low temperature stress. MGT, Mean Germination Time; GR, Germination Rate GP, Germination Percentage; SL, Shoot Length; RL, Root Length; PL, Plumule Length; RSR, Root-to-Shoot Ratio; SVI, Seedling Vigor Index.

Principal Component Analysis (PCA) was performed to explore the relationships between genotypes and the measured traits under low temperature stress (24 °C/12 °C). The first two principal components explained 83.30% of the total variance, with PC1 accounting for 52.50% and PC2 accounting for 30.80%, indicating that the first two components captured a substantial proportion of the variability observed among the genotypes (**Figure 8**).

The PCA biplot clearly separated the genotypes into three distinct groups (G1, G2, and G3) according to their trait associations. Group G1 is located on the positive side of PC1 and includes the genotypes, ML13, ML2-72, and ML2-37.These genotypes are positioned in the direction of the vectors corresponding to RL, GP, SL, PL, SVI, RSR, and GR, indicating strong positive associations with these traits. The proximity of these genotypes to these vectors suggests that they exhibit higher germination performance and seedling vigor under low temperature stress. These associations suggest that these genotypes exhibited relatively better germination performance and seedling vigor under low-temperature conditions. Group G2 is represented by a single genotype US1-2. This genotype is located in the upper part of the biplot and shows a strong association with mean germination time (MGT). This indicates that its germination behavior is mainly influenced by this parameter, distinguishing it from the other genotypes by its high sensitivity across the severe low temperature stress. Group G3 is located on the negative side of PC1 and includes, ML2-5, ML2-10.These genotypes are positioned opposite to the vectors associated with the dominant traits of G1. This position suggests that they exhibit lower values for GP, RL, SVI, and other vigor related traits, indicating an intermediate sensitivity to low temperature stress during germination compared to the other genotypes, except for US1-2.

Overall, the PCA analysis revealed a clear structuring of genotypes into three groups according to their germination and seedling growth responses under low temperature conditions. Traits such as GP, RL, SL, PL, and SVI contributed strongly to the observed variation among genotypes and may represent useful indicators of seedling performance under low-temperature conditions.

The genotypes ML13, ML2-72, and ML2–37 were associated with more favorable germination and seedling growth traits under low-temperature conditions, suggesting relatively better adaptation during early developmental stages. In contrast, ML2–5 and ML2–10 appeared more sensitive to cold stress, while US1–2 is the most sensitive genotype.

## 4 Discussion

The results of this study clearly demonstrate the significant effect of low-temperature stress on germination and early seedling-growth in sesame. However, a significant genotypic variation among sesame genotypes was found in response to this stress. The differentiation between the potentially cold-tolerant genotypes (ML2-37, ML2-72) and sensitive (US1-2, ML2-5) genotypes became evident as temperatures dropped to 24 °C/12 °C. While standard sesame cultivars typically require temperatures above 20 °C for optimal germination (El Harfi et al., 2016), the identified mutants maintained germination percentages exceeding 85% under cooler conditions. This finding aligns with the positive correlation between germination success and cold tolerance reported in other warm season crops (Yousefi et al., 2020). The high germination rate and short mean germination time observed in promising tolerant mutants could be associated with physiological and biochemical mechanisms involved in cold stress tolerance. According to (Mavi et al., 2010), seeds that germinate quickly under stress are better able to preserve protein and DNA integrity, thereby initiating metabolic activity before secondary damage accumulates. Early seedling growth, particularly root development, is a critical determinant of crop establishment. Our data showed that cold stress significantly inhibited root and shoot elongation, which is consistent with findings by (Abbas et al., 2025). However, the tolerant mutants ML2–37 and ML2–72 maintained higher plumule length and superior root-to-shoot ratio at the lowest temperature stress. A vigorous root system under cold stress is essential for water and nutrient uptake when soil hydraulic conductivity is low (Hund, 2003). The SVI emerged as a robust indicator of cold tolerance, effectively summarizing the cumulative effects of stress on both germination and early-growth. The drastic reduction in SVI for ML2–5 highlights that is more closely associated with the stress regime and reduced performance, whereas the stability of ML2–37 suggests high potential for field establishment under such a condition. In addition, Duncan’s multiple range test revealed that US1–2 was significantly separated from the other genotypes in most cases, forming a distinct statistical group across temperature regimes and evaluated traits. Its consistently lower germination and seedling growth performance under low-temperature conditions indicated that US1–2 was the most cold-sensitive genotype among the materials evaluated in this study.

Previous studies have suggested that cold-tolerant plants may maintain membrane fluidity by increasing the proportion of unsaturated fatty acids (Murata and Los, 1997). It is, thus, hypothesized that mutants ML2–37 and ML2–72 might possess alterations in fatty acid desaturase activity, allowing them to maintain membrane integrity under cool conditions. Also, the accumulation of osmoprotectants such as proline, glycine betaine, and soluble sugars is a universal response to cold stress (Hare et al., 1998). These solutes stabilize proteins and membranes and lower the freezing point of the cytoplasm Abbas et al. (2025) reported significantly higher proline and sugar accumulation in cold-tolerant sesame genotypes at 14 °C. Accordingly, the superior performance of our cold tolerant mutants could be associated with efficient osmotic adjustment. Likewise, cold tolerant genotypes would have exhibited enhanced activity of antioxidant enzymes (SOD, CAT, POX) to scavenge reactive oxygen species (ROS) induced by cold stress (Abbas et al., 2025).

The correlation and principal component analyses performed in this study provided a comprehensive multivariate framework to identify key traits associated with sesame tolerance to low temperature during germination and early seedling development. Significant relationships were observed among several germination and seedling growth traits under low temperature stress (24 °C/12 °C), highlighting the coordinated physiological responses involved in cold tolerance (**Figure 8**). Similar associations among germination traits and seedling vigor parameters have been reported in sesame and other crops (Abbas et al., 2025; Kouighat et al., 2023; Yousefi et al., 2020).

In the present study, traits including SVI, GP, GR, MGT, RL, SL, PL and RSR emerged as relevant selection criteria. Strong positive correlations were observed among germination percentage (GP), seed vigor index (SVI), shoot length (SL), plumule length (PL), and root length (RL), indicating that genotypes exhibiting higher germination capacity also tend to develop more vigorous seedlings under low temperature conditions. These results suggest that rapid germination is closely linked to improved early seedling growth, which a crucial adaptive strategy is allowing plants to establish successfully under unfavorable thermal environments. Similar relationships between germination performance and seedling vigor have been reported in several crops exposed to cold stress, where vigorous seedlings are often associated with improved stress resilience during early developmental stages (Bewley and Black, 1994; Hasanuzzaman et al., 2013).

Conversely, mean germination time (MGT) was negatively associated with germination rate (GR) and germination percentage (GP), indicating that genotypes characterized by faster germination tend to exhibit lower germination time and enhanced seedling vigor. This negative relationship reflects the physiological advantage of rapid germination under stress conditions, as shorter germination time reduces the exposure of seeds to unfavorable environmental factors and promotes faster seedling establishment.

PCA biplot analysis further identified three distinct genotype clusters, with group G1 including ML13, ML2-72, and ML2-37, representing the severe low temperature tolerant genotypes, group G2 is US1–2 representing the highest sensitive genotype, and group G3 identified by ML2–5 and ML2-10, exhibiting moderate tolerance to low temperature stress. The identification of ML2–37 and ML2–72 as a genotypes tolerant to severe temperature stress, has significant implications for sesame breeding in Morocco. The current reliance on June sowing exposes the crop to peak summer water deficits. By utilizing these cold-tolerant mutants, breeders can develop cultivars suitable for early-spring sowing in April. This shift would align the crop’s vegetative phase with spring rainfall, reducing the irrigation water footprint. Furthermore, since these mutants were previously characterized for drought tolerance (Kouighat et al., 2023), they represent multi-stress tolerant germplasm ideal for the unpredictable climate of semi-arid regions. Breeding strategies should focus on the introgression of these tolerance traits into high-yielding agronomic backgrounds through controlled crosses and developing molecular markers to facilitate selection. However, several limitations of this study must be acknowledged. First, the evaluation was conducted under controlled laboratory conditions (Petri dishes), which provide a homogeneous environment that does not capture the complexity of field conditions. In the field, germinating seeds face heterogeneous soil structures, soil-borne pathogens, and fluctuating temperatures (including day/night amplitude) that can interact with cold stress to affect plantlet establishment. Second, the stress was applied in isolation; however, in real field conditions, cold stress often co-occurs with transient waterlogging or drought.

Future research must therefore prioritize extensive field validation. Hence, multi-location trials across Morocco’s agro-ecological zones (Tadla, Saïs, Gharb) over at least three seasons are essential to confirm whether the lab observed tolerance translates into better seed germination and plantlet establishment and, ultimately, improved yield under early planting field conditions. Additionally, biochemical profiling (proline, MDA, antioxidant enzymes) and molecular characterization (transcriptomics, genome resequencing) are needed to definitively identify the causal mechanisms and specific mutations conferring tolerance.

## 5 Conclusions

This study successfully identified significant genotypic variation among the investigated sesame genotypes, including the EMS-induced mutants, in response to progressive low temperature stress during germination. Under controlled conditions simulating early spring temperatures in Morocco (24 °C/12 °C night minima), mutants ML2–37 and ML2–72 demonstrated superior cold tolerance compared to the other mutants. They exhibited high germination percentages (>85%), rapid germination rates, superior plumule length and robust seedling vigor even under the coldest regime. In contrast, genotype US1–2 was identified as highly sensitive, making it unsuitable for early sowing.

The present study provides new insights into the response of EMS-induced sesame mutants to moderate cold temperature stress during germination and early seedling development under conditions representative of Moroccan spring sowing environments. The results suggest that mutants ML2–37 and ML2–72 could constitute promising genetic resources for breeding programs aimed at improving sesame adaptation to early sowing and water-limited environments. However, these findings should be interpreted with caution, as all experiments were conducted under controlled *in vitro* conditions that do not fully reproduce the complexity of field environments, including soil heterogeneity, fluctuating climatic factors, and biotic interactions. Therefore, further field evaluations across multiple environments and seasons are necessary to confirm the agronomic performance, stability, and practical value of these mutant lines under real cultivation conditions.

## Data Availability

The datasets generated in this study are available from INRA (Morocco) and can be requested from the corresponding author or the institution upon reasonable request.
